# 转录因子EB相关自噬在多发性骨髓瘤治疗中的作用及其机制研究

**DOI:** 10.3760/cma.j.issn.0253-2727.2021.05.010

**Published:** 2021-05

**Authors:** 志华 张, 荣娟 张, 凝 韩, 冲 栗, 丽红 王, 恩鸿 邢, 翠红 谷, 长来 郝

**Affiliations:** 承德医学院附属医院 067000 Affiliated Hospital of Chengde Medical College, Chengde 067000, China

**Keywords:** 多发性骨髓瘤, 自噬, TFEB, 硼替佐米, 西拉美新, Multiple myeloma, Autophagy, TFEB, Bortezomib, Siramesine

## Abstract

**目的:**

明确硼替佐米和（或）西拉美新作用于多发性骨髓瘤（MM）细胞株后细胞增殖、转录因子EB（TFEB）核转位表达变化及自噬水平，为进一步探讨TFEB对自噬的调控机制提供依据。

**方法:**

体外培养MM细胞株RPMI8226及U266，并以一定浓度的硼替佐米和西拉美新处理MM细胞，CCK-8法检测细胞增殖，实时定量PCR和Western blot法检测TFEB、自噬相关因子LC3B、Beclin1、p62、LAMP1的mRNA和蛋白相对表达量。

**结果:**

随着硼替佐米浓度增加及作用时间延长，两个细胞系的增殖抑制率增高（*P*<0.05）。硼替佐米和西拉美新联用对上述MM细胞株的增殖有协同抑制作用（*P*<0.05）。空白对照组、单药组、联合用药组处理MM细胞株后，细胞质中TFEB的mRNA和蛋白相对表达量依次下降（*P*<0.05），细胞核中TFEB的mRNA和蛋白相对表达量依次上升（*P*<0.05），自噬相关因子LC3B、Beclin1、LAMP1的mRNA和蛋白相对表达量依次上升，p62的mRNA和蛋白相对表达量依次下降（*P*<0.05）。

**结论:**

硼替佐米和西拉美新具有协同抑制MM细胞增殖作用，与其诱导MM细胞株自噬表达增强相关，发生核转位的TFEB表达亦增强。

## Abstract (en)

**Objective:**

To clarify the effects of bortezomib combined with or without siramesine on the proliferation of multiple myeloma cell lines, the expression changes of transcription factor EBC（TFEB）nuclear translocation and the level of autophagy, and to provide basis for further exploring the regulation mechanism of transcription factor TFEB on autophagy.

**Methods:**

The multiple myeloma cell lines RPMI8226 and U266 were cultured in vitro, and the multiple myeloma cells were treated with a certain concentration of bortezomib and siramesine. The changes of cell proliferation inhibition were detected by CCK-8 method. Real time PCR and Western blot were used to detect the relative expression of TFEB, autophagy-related factor LC3B, Beclin1, p62, LAMP1 mRNA and protein.

**Results:**

As the concentration of bortezomib increased and the duration of action increased, the proliferation inhibition rates of the two cell lines gradually increased（*P*<0.05）. The combination of the two drugs has a synergistic inhibitory effect on the proliferation of the above-mentioned multiple myeloma cell lines（*P*<0.05）. In the blank control group, single drug group, and combination drug group, the relative expression of TFEB mRNA and protein in the cytoplasm decreased sequentially （*P*<0.05）, and the relative expression of TFEB mRNA and protein in the nucleus increased sequentially（*P*<0.05）. The relative expression of autophagy-related factors LC3B, Beclin1, LAMP1 mRNA and protein increased sequentially, and the relative expression of p62 mRNA and protein decreased sequentially（*P*<0.05）.

**Conclusion:**

Bortezomib and siramesine can synergistically inhibit the growth of multiple myeloma cells, which is related to the increased autophagy expression in multiple myeloma cell lines and the expression of TFEB with nuclear translocation is also enhanced.

多发性骨髓瘤（MM）是一种起源于B细胞系并能产生单克隆免疫球蛋白的恶性增殖性疾病，约占血液系统恶性肿瘤的13％，占所有恶性肿瘤的1％[1]。自噬是真核细胞降解和回收受损细胞器和生物大分子的过程。自噬作为一种重要的生理与病理机制，与疾病的发生、发展密切相关。转录因子EB（transcription factor EB, TFEB）是调控自噬的关键转录分子[2]–[5]，其核转位能整合与协调细胞系统降解和能量回收[6]，是细胞压力和能量代谢的关键分子事件[7]。硼替佐米作为一种蛋白酶体抑制剂，能选择性抑制26S蛋白酶体，有效抑制NF-κB的活性及细胞增殖相关基因表达，进而诱导MM细胞凋亡[8]，在MM治疗中取得了令人瞩目的疗效。然而，临床在应用硼替佐米的过程中，多数患者在疾病后期不可避免地面临不能耐受的不良反应及药物耐药[9]。寻找一种新型药物与硼替佐米联合使用以降低硼替佐米的不良反应、克服其耐药性尤为迫切。亲溶酶体剂西拉美新是一类具有碱性pK值和较长疏水链的胺类化合物，经细胞吞饮作用可进入细胞并聚集在溶酶体中，达到一定程度时可使溶酶体膜破裂，释放水解酶，导致细胞水解和死亡[10]。本研究将硼替佐米与西拉美新联合作用于MM细胞株，观察TFEB在细胞核、细胞质中的表达水平及自噬相关因子表达水平变化，进一步探讨TFEB相关的自噬在MM治疗中的作用及其机制，为硼替佐米更好地治疗MM提供新的思路及理论依据。

## 材料与方法

1. 材料和试剂：RPMI8226及U266细胞购自上海中美合资博慧斯生物医药科技有限公司，硼替佐米（规格5 mg/瓶，型号S1013）购自美国Selleck公司，Siramesine fumarate salt（规格25mg/瓶，型号SML0976）购自美国Sigma公司。兔抗人LC3B、Beclin-1、LAMP1、p62、TFEB单克隆抗体购自英国Abcam公司，核蛋白和胞质蛋白提取试剂盒购自上海BestBio生物有限公司，TRIzol试剂购自美国Invitrogen公司，反转录试剂盒及引物均为美国Invitrogen公司产品。β-actin单克隆抗体为美国Santa Cruz Biotechnlogy公司产品，羊抗兔二抗购自美国KPL公司。实时定量PCR仪为美国Invitrogen公司产品，DYY-6C电泳仪为北京六一生物科技有限公司产品。

2. 细胞培养：RPMI8226及U266细胞培养于含青霉素（100 U/ml）、链霉素（100 g/ml）、10％胎牛血清的RPMI 1640培养基中，置于饱和湿度、37°C、5％CO_2_培养箱中常规传代培养，取对数生长期细胞用于后续实验。

3. CCK-8法检测不同药物对细胞增殖的影响：取对数生长期细胞，以8×10^3^/孔的密度接种于96孔板内，实验组加入特定浓度的硼替佐米（3、6、9、12 nmol/L）、西拉美新（0.5、1.0 µmol/L）及以上两种药物。对照组以RPMI1640完全培养基代替药物。置于37°C培养箱培养相应时间后，每孔加入10 µl CCK-8溶液（避免产生气泡），将96孔板置于37°C培养箱中孵育2 h后置于酶标仪中测定其在450 nm处的吸光度（*A*），按下式计算细胞增殖抑制率（％）＝（*A*c−*A*s）/（*A*c−*A*b）×100％；*A*s：实验组（药物2 µl、细胞悬液98 µl、CCK-8 10 µl），*A*c：对照组（细胞悬液100 µl、CCK-8 10 µl），*A*b：空白组（培养基100 µl、CCK-8 10 µl）。

4. 蛋白及RNA的提取：收集各组细胞，应用核蛋白/胞质蛋白提取试剂盒分别提取胞质蛋白与核蛋白，利用PARIS Kit核酸纯化试剂盒提取各实验组细胞的胞质RNA和核RNA。

5. Western blot法检测细胞核和细胞质LC3B、Beclin-1、LAMP1、p62、TFEB蛋白的表达量：实验分为对照组、硼替佐米（6 nmol/L）组、西拉美新（1 µmol/L）组和硼替佐米（6 nmol/L）与西拉美新（1 µmol/L）联合组。收集各组细胞，用组织快速裂解液裂解细胞后提取总蛋白质，用二喹啉甲酸（BCA）法测定蛋白浓度。取50 µg蛋白行120 g/L、50 g/L SDS聚丙烯酰胺凝胶电泳，电转移至PVDF膜上，用50 g/L脱脂奶粉溶液室温封闭2 h后加入兔抗人LC3B（稀释浓度为1∶5000）、Beclin-1（稀释浓度为1∶5000）、LAMP1（稀释浓度为1∶5000）、p62（稀释浓度为1∶5000）、TFEB（细胞核和细胞质，稀释浓度为1∶1000）一抗，4°C摇床过夜，室温下洗膜3次，加入辣根过氧化物酶标记的羊抗兔二抗（稀释浓度为1∶10 000）孵育2 h，室温下洗膜，电化学发光超敏发光液显影，暗室曝光。采用成像系统进行蛋白表达检测，以LC3B、Beclin-1、LAMP1、p62、TFEB（细胞核和细胞质）与β-actin或GAPDH条带的*A*值比值表示LC3B、Beclin-1、LAMP1、p62、TFEB（细胞核和细胞质）蛋白的相对表达水平。

6. 实时定量PCR法检测LC3B、Beclin-1、LAMP1、p62、TFEB（细胞核和细胞质）的mRNA表达水平：实验分组同Western blot法，培养24 h后收集细胞。用TRIzol提取液提取总RNA，按照反转录试剂盒说明书合成cDNA。以cDNA为模板，分别用目的基因LC3B、Beclin-1、LAMP1、p62引物和内参基因β-actin引物，目的基因TFEB引物和内参基因GAPDH引物对目的片段进行扩增，经预变性、变性、退火及延伸等步骤，绘制熔解曲线，读取Ct值，以2^−ΔΔCt^计算实验结果。各因子引物序列见表1。

**表1:** Beclin-1、LC3B、p62、LAMP1、TFEB、GAPDH引物序列及扩增片段大小

因子	引物序列	扩增片段大小（bp）
Beclin1	上游 5′-CCAGATGCGTTATGCCCAGAC-3′	149
	下游 5′-CATTCCATTCCACGGGGAACAC-3′	
LC3B	上游 5′-AGTTGGCAAACGCAGGGTA-3′	80
	下游 5′-TTAGGAGTCAGGGACCTTCAGCA-3′	
p62	上游 5′-ACATAGCTTGCCTAATGGCTTTCAC-3′	143
	下游 5-CCTGCCTGCTGACAACACCTA-3′	
LAMP1	上游5-CACGTTACAGCGTCCAGCTCA-3′	184
	下游5-AGCGTTACGGTCACGTTGTTCA-3′	
TFEB	上游5′-GGCAACAGTGCTCCCAATAG-3′	138
	下游5′-GCATCTGCATTTCAGGATTGATG-3′	
GAPDH	上游5′-GCACCGTCAAGGCTGAGAAC-3′	138
	下游5′-TGGTGAAGACGCCAGTGGA-3′	

7. 统计学处理：采用SPSS 19.0软件进行统计学分析，计量资料以均数±标准差表示，CCK-8实验结果行*t*检验和*t′*检验。Western blot和实时定量PCR实验结果行单因素方差分析，用LSD-t法行组间比较，*P*<0.05为差异有统计学意义。两药联合作用效果应用析因设计方法。

## 结果

一、CCK-8法检测不同药物对细胞增殖的影响

1. 硼替佐米对MM细胞活性的影响：3、6、9、12 nmol/L硼替佐米处理RPMI8226和U266细胞株6、12、24 h后细胞的增殖抑制率见表2、3，结果显示，除在细胞系U266中硼替佐米浓度6 nmol/L时不同作用时间下增殖抑制率无明显变化（*P*>0.05）外，随着硼替佐米浓度增加及处理时间延长，两个细胞系的增殖抑制率逐渐增高（*P*<0.001），硼替佐米对MM细胞系的增殖抑制作用呈时间和浓度依赖性。

**表2:** CCK-8法检测不同浓度硼替佐米处理RPMI8226细胞的不同时间增殖抑制率（％，*x*±*s*）（每组设5个复孔，实验重复3次）

作用时间	硼替佐米浓度（nmol/L）				
	3	6	9	12	*P*值
6 h	1.00±0.12	3.42±0.01	5.40±0.36	9.40±0.17	<0.001
12 h	2.30±0.96	4.17±0.04	10.19±0.04	14.51±0.07	<0.001
24 h	6.49±0.03	12.75±0.07	17.93±0.04	29.56±0.01	<0.001

*P*值	<0.001	<0.001	<0.001	<0.001

**表3:** CCK-8法检测不同浓度硼替佐米处理U266细胞的不同时间增殖抑制率（％，*x*±*s*）（每组设5个复孔，实验重复3次）

作用时间	硼替佐米浓度（nmol/L）				
	3	6	9	12	*P*值
6 h	1.10±0.06	7.06±0.36	14.40±0.40	15.91±0.01	<0.001
12 h	2.00±0.56	10.90±3.34	16.11±0.35	20.03±0.71	<0.001
24 h	7.14±0.12	13.62±3.09	20.05±0.03	33.43±0.11	<0.001

*P*值	<0.001	0.283	<0.001	<0.001

2. 两药联合对MM细胞活性的影响：两药物以不同浓度梯度联合作用于MM细胞系24 h，增殖抑制率见表4、5。应用两因素析因设计方法分析两药联合作用，我们发现硼替佐米和西拉美新联用对上述MM细胞株的增殖有协同抑制作用（*P*<0.001）（表6、7）。为观察自噬，我们选取6 nmol/L硼替佐米、1 µmol/L西拉美新作用24 h用于后续实验。

**表4:** CCK-8法检测不同浓度硼替佐米联合西拉美新处理RPMI8226细胞24 h的增殖抑制率（％，*x*±*s*）（每组设5个复孔，实验重复3次）

西拉美新浓度（µmol/L）	硼替佐米浓度（nmol/L）			
	3	6	9	12
0.5	14.52±0.03	26.11±0.03	31.15±0.78	41.83±0.04
1.0	25.47±0.03	33.56±0.01	38.22±0.03	50.29±0.09

**表5:** CCK-8法检测不同浓度硼替佐米联合西拉美新处理U266细胞24 h的增殖抑制率（％，*x*±*s*）（每组设5个复孔，实验重复3次）

西拉美新浓度（µmol/L）	硼替佐米浓度（nmol/L）			
	3	6	9	12
0.5	19.44±0.20	30.69±0.27	34.15±0.27	46.23±0.01
1.0	25.38±0.06	35.38±0.25	40.52±0.45	55.47±0.03

**表6:** 不同浓度硼替佐米联合西拉美新处理RPMI8226细胞24 h对增殖影响的两因素析因设计方差结果

方差来源	离均差平方和	自由度	均方	*F*值	*P*值
硼替佐米（A）	2112.21	3	704.07	8980.02	<0.001
西拉美新（B）	431.80	1	431.80	5507.38	<0.001
交互作用（A×B）	13.71	3	4.57	58.29	<0.001
误差	1.254	16	0.078	−	−

合计	28133.07	24	−	−	−

注：−：无数据

**表7:** 不同浓度硼替佐米联合西拉美新处理U266细胞24 h对增殖影响的两因素析因设计方差结果

方差来源	离均差平方和	自由度	均方	*F*值	*P*值
硼替佐米（A）	2494.47	3	831.49	17240.4	<0.001
					
西拉美新（B）	258.14	1	258.14	5352.28	<0.001
交互作用（A×B）	16.65	3	5.55	115.09	<0.001
误差	0.770	16	0.048	−	−

合计	33713.68	24	−	−	−

注：−：无数据

二、实时定量PCR法和Western blot法分析硼替佐米、西拉美新及两药联合对RPMI8226和U266细胞株自噬相关因子mRNA和蛋白表达的影响

1. 硼替佐米单药对MM细胞株自噬相关因子mRNA和蛋白表达的影响：硼替佐米（6 nmol/L）处理RPMI8226和U266细胞株24 h后，与对照组比较，硼替佐米组自噬相关因子LC3B、Beclin-1、LAMP1的mRNA和蛋白表达水平上升，p62的mRNA和蛋白表达水平下降，即硼替佐米可促进RPMI8226和U266细胞株自噬表达增加（图1～3）。

**图1:**
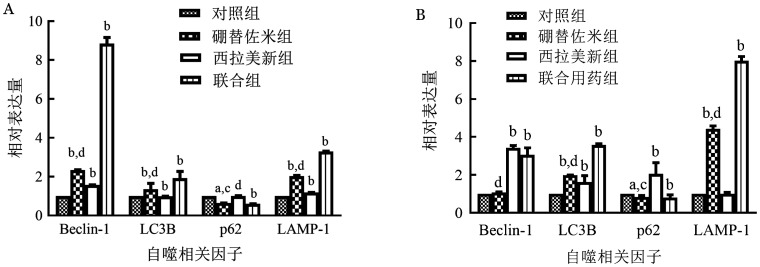
实时定量PCR法检测不同药物处理RPMI8226（A）和U266（B）细胞系24 h对自噬相关因子表达的影响 实验重复3次；与对照组相比，^a^*P*<0.05，^b^*P*<0.001；与联合用药组相比，^c^*P*<0.05，^d^*P*<0.001

**图2:**
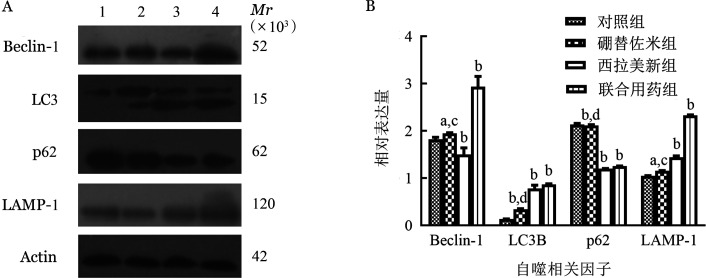
Western blot法检测不同药物处理RPMI8226细胞系24 h自噬相关因子蛋白表达条带（A）和蛋白相对表达量（B） A：1：对照组；2：硼替佐米组；3：西拉美新组；4：联合用药组；B：实验重复3次；与对照组相比，^a^*P*<0.05，^b^*P*<0.001；与联合用药组相比，^c^*P*<0.05，^d^*P*<0.001

**图3:**
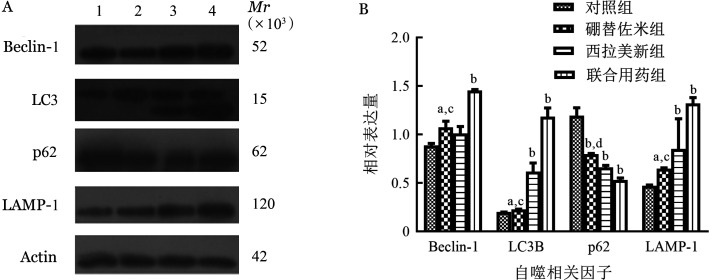
Western blot法检测不同药物处理U266细胞系24 h自噬相关因子蛋白表达条带（A）和蛋白相对表达量（B） A：1：对照组；2：硼替佐米组；3：西拉美新组；4：联合用药组；B：实验重复3次；与对照组相比，^a^*P*<0.05，^b^*P*<0.001；与联合用药组相比，^c^*P*<0.05，^d^*P*<0.001

2. 硼替佐米联合西拉美新对MM细胞株自噬相关因子mRNA和蛋白表达的影响：联合用药组与单药组比较，自噬相关因子LC3B、Beclin-1、LAMP1的mRNA和蛋白表达增加，p62的mRNA和蛋白表达量下降，各组间差异有统计学意义（*P*<0.05），即联合用药组较单药组自噬表达量增加（图1～3）。

三、实时定量PCR和Western blot法分析硼替佐米、西拉美新及两药联合对RPMI8226和U266细胞株细胞核和细胞质中TFEB mRNA和蛋白表达的影响

1. 硼替佐米单药对MM细胞株细胞核和细胞质中TFEB mRNA和蛋白表达的影响：硼替佐米（6 nmo/L）处理RPMI8226和U266细胞株24 h后，与空白对照组比较，硼替佐米组TFEB在细胞质中的表达量下降，在细胞核中的表达量上升，组间比较差异有统计学意义（*P*<0.05），即硼替佐米可促进RPMI8226和U266细胞株TFEB核转移（图4～6）。

**图4:**
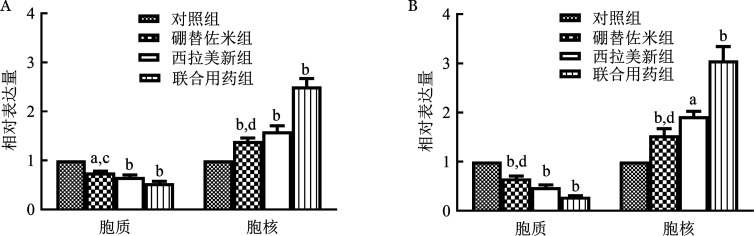
实时定量PCR法检测不同药物处理RPMI8226（A）和U266（B）细胞系24 h对细胞质、细胞核TFEB mRNA表达的影响 实验重复3次；与对照组相比，^a^*P*<0.05，^b^*P*<0.001；与联合用药组相比，^c^*P*<0.05，^d^*P*<0.001

**图5:**
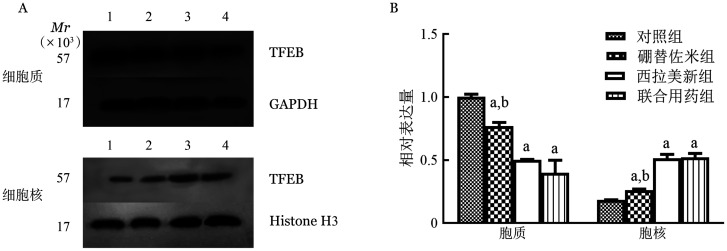
Western blot法检测不同药物处理RPMI8226细胞系24 h TFEB细胞质、细胞核蛋白表达条带（A）和蛋白相对表达量（B） A：1：对照组；2：硼替佐米组；3：西拉美新组；4：联合用药组；GADPH：甘油醛-3-磷酸脱氢酶内参；Histone H3：组蛋白H3内参；B：实验重复3次；与对照组相比，^a^*P*<0.001；与联合用药组相比，^b^*P*<0.001

**图6:**
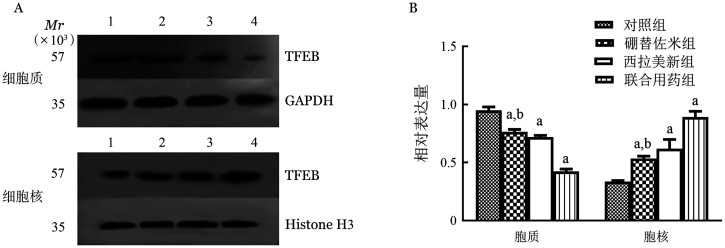
Western blot法检测不同药物处理U266细胞系24 h细胞质、细胞核中TFEB蛋白表达条带（A）和蛋白相对表达量（B） A：1：对照组；2：硼替佐米组；3：西拉美新组；4：联合用药组；GADPH：甘油醛-3-磷酸脱氢酶内参；Histone H3：组蛋白H3内参；B：实验重复3次；与对照组相比，^a^*P*<0.001；与联合用药组相比，^b^*P*<0.001

2. 硼替佐米联合西拉美新对MM细胞株细胞核和细胞质中TFEB mRNA和蛋白表达的影响：联合用药组与单药组比较，TFEB在细胞质中的表达量下降，在细胞核中的表达量上升，组间比较差异有统计学意义（*P*<0.05），即两药联合可进一步促进RPMI8226和U266细胞株TFEB核转移（图4～6）。

## 讨论

MM是终末B淋巴细胞恶性增殖性肿瘤，以分泌单克隆免疫球蛋白为主要特点[11]，临床以骨痛、贫血、出血等为主要表现[12]。近几年来，随着蛋白酶体抑制剂等新型药物的问世，MM的治疗取得了极大的进步[13]。但耐药使其呈现出复发难治的特点，MM仍是血液系统肿瘤研究的难点和热点。

自噬是真核细胞用于降解和回收利用细胞内受损细胞器和生物大分子的过程，对细胞自身稳定的维持发挥着重要作用[14]。目前研究表明，自噬具有双重作用，一方面自噬可以实现体内多余物质的清除和能量的供应，达到维持细胞稳态的效果；另一方面，过度自噬又可导致细胞发生“自噬性死亡”。其中，LC3B、Becline-1、p62、LAMP-1分别参与自噬形成过程，故本文通过检测上述四个指标变化间接反映自噬水平。

TFEB是小眼畸形相关转录因子家族成员之一[15]–[18]。最近研究表明TFEB与溶酶体生成及自噬关系密切[19]。TFEB对自噬功能的调节依赖其核转位的增加。在细胞核中，TFEB激活CLEAR网络基因的表达[20]，并能转录调控其下游细胞自噬和溶酶体相关基因表达，调节多个自噬的关键环节。TFEB的核转位及表达变化引起的自噬水平改变参与了多种疾病的发生、发展、预后和转归[21]–[23]。

硼替佐米的主要作用机制为选择性抑制26S蛋白酶体，减少细胞核因子NF-κB抑制因子（IκB）的降解。随着研究的深入，越来越多的研究表明NF-κB与自噬存在关联[24]–[25]。为了揭示TFEB对自噬调节的机制，本研究在硼替佐米导致TFEB水平变化的基础上，检测了自噬表达水平。

我们将不同浓度的硼替佐米作用于MM细胞株不同时间，发现随着硼替佐米浓度的增加及处理时间的延长，两个细胞系的增殖抑制率逐渐增高。我们选取浓度为6 nmol/L的硼替佐米处理MM细胞系24 h，结果显示，与空白对照组相比，硼替佐米组细胞核中TFEB mRNA和蛋白的表达量增加，细胞质中的表达量减少。自噬相关因子LC3B、Beclin-1、LAMP1的表达升高，p62的表达下降，硼替佐米促进了MM细胞株自噬的表达，与Vogl等[26]的研究一致。

然而，硼替佐米不良反应和耐药性的出现使MM治疗面临严峻挑战。亲溶酶体剂西拉美新是一种σ-2受体激动剂，最初作为抗抑郁药应用于临床，但由于抗抑郁作用轻微而被遗弃。近年来研究表明，其可通过改变溶酶体膜的通透性，使溶酶体酸性物质释放导致细胞死亡，达到抗肿瘤作用[27]–[29]。于是我们希望验证西拉美新能否通过影响溶酶体功能抑制TFEB核易位，降低自噬表达，进而提高硼替佐米对MM的敏感性。

本研究应用析因设计实验方法分析西拉美新与硼替佐米联合应用对细胞增殖的抑制作用。结果显示，联合用药对MM细胞株的增殖表现为协同抑制作用。为进一步探讨其细胞毒性的作用机制，我们分别从基因和蛋白水平研究了硼替佐米联合或不联合西拉美新对MM细胞株TFEB表达水平及自噬表达水平的影响。结果显示，与单药组比较，联合用药组自噬表达进一步增强，此时发生核转位的TFEB表达进一步增多，与我们预期的结果相反。可能有如下原因：①大部分文献中提到硼替佐米可使MM细胞自噬表达增多均针对硼替佐米耐药细胞株，而本研究应用非耐药细胞株，还需构建硼替佐米耐药细胞株进行进一步研究；②西拉美新的作用机制仍待进一步完善：西拉美新诱导的MM细胞死亡可能通过增加溶酶体膜通透性，使其产生脂质过氧化作用[30]，也有研究表明西拉美新可通过多种机制、多种信号通路达到良好的抗肿瘤作用（诱导caspase-3的激活，促进细胞凋亡；诱导自噬，导致死亡；影响细胞周期等）[31]。

总之，我们的研究证实硼替佐米对MM细胞株具有增殖抑制作用，与硼替佐米诱导的MM细胞株自噬表达增强相关；硼替佐米联合西拉美新对MM细胞株增殖具有协同抑制作用，与西拉美新诱导的硼替佐米自噬表达进一步增强相关，发生核转位的TFEB进一步增加，上述结论为硼替佐米更好地治疗MM提供了新思路。
